# Ataxia due to vitamin E deficiency: A case report and updated review

**DOI:** 10.1002/ccr3.6303

**Published:** 2022-09-06

**Authors:** Sangharsha Thapa, Sangam Shah, Swati Chand, Sanjit Kumar Sah, Pawan Gyawali, Sandip Paudel, Pitambar Khanal

**Affiliations:** ^1^ University of Minnesota Medical School Twin Cities Minneapolis Minnesota USA; ^2^ Institute of Medicine Tribhuvan University Kirtipur Nepal; ^3^ Rochester General Hospital Rochester New York USA

**Keywords:** ataxia, AVED, vitamin E

## Abstract

Ataxia with vitamin E deficiency (AVED) is a rare cause of hereditary ataxia in developing countries with unknown prevalence. AVED is an autosomal‐recessive disorder, which is characterized by ataxia, areflexia, and proprioceptive and vibratory sensory loss. The disease is characterized clinically by symptoms with often resembling to those of Friedreich ataxia (FRDA). Vitamin E supplementation improves symptoms and prevents the progression of the disease. In this case report, we reviewed the recently updated findings in AVED in regard to the management and present a case of AVED in a 16‐year‐old boy, who was initially misdiagnosed as FRDA, prior to the genetic test.

## 1 INTRODUCTION

**FIGURE 1:**
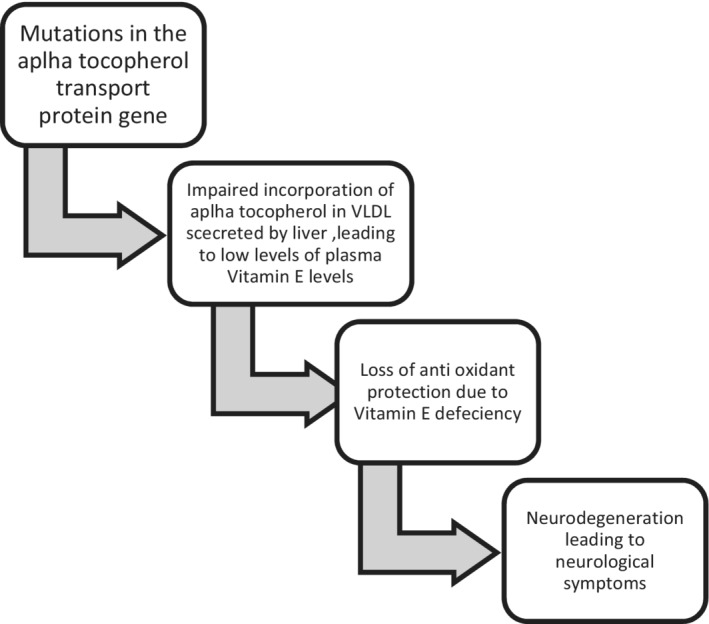
Pathogenesis of ataxia due to vitamin E deficiency

Ataxia with isolated vitamin E deficiency (AVED) is a rare autosomal‐recessive disease that can result from a mutation in the alpha‐tocopherol transfer protein gene on chromosome 8. Abetalipoproteinemia also known as bassen–Kornzweig disease can also lead to hereditary vitamin E deficiency due to the abnormal absorption of the fat‐soluble vitamins including vitamin E. Ataxia with isolated vitamin E deficiency is characterized by progressive cerebellar ataxia, dysarthria, and progressive clumsiness. AVED usually manifests in the late childhood or early teens with a clinical picture similar to Friedreich ataxia.^1^ Although AVED and FRAD are clinically similar, there are some clinical features which are specific to the respective disease. Skeletal deformities like scoliosis and pes cavus are present in Friedreich ataxia. Meanwhile, the presence of head titubation or dystonia with fewer effects on the heart is seen associated in AVED. Hereditary ataxia can be inherited in autosomal dominant, autosomal recessive, and X‐linked manner. Autosomal recessive is the most common inheritance pattern and called as recessive ataxia or spinocerebellar ataxia. There are five typical autosomal‐recessive disorders in which ataxia is a prominent feature: Friedreich ataxia, ataxia‐telangiectasia, ataxia with vitamin E deficiency, ataxia with oculomotor apraxia (AOA), and spastic ataxia^2^, ^3^ The prevalence of AVED is unknown but it is low, and is documented more frequently from North African counties. The pathogenesis with regard to genetic abnormalities and effect on nervous systems has been described below (Figure 1).

Vitamin E is a potent lipid‐soluble antioxidant, loss of which leads to neurological symptoms. The exclusion of AVED from nongenetic causes of vitamin E deficiency is required for the definitive management. All forms of pediatric ataxia should be tested for AVED with vitamin E levels, to make an early diagnosis and prevent the patient from a delirious outcome with definitive management. Here, we present a case of a 16‐year boy, who was initially managed as Friedreich ataxia prior to the genetic test on the basis of clinical picture but later found to have AVED.

## 2 CASE PRESENTATION

A 16‐year‐old boy was referred to the tertiary neurology center for assessment of his progressive ataxia and generalized weakness of the body. His first symptoms started when he was 10 years old with progressive gait difficulties and generalized weakness of the body. His gait difficulties were associated with wasting of his muscles and slurring of speech. Pregnancy and birth history were normal. Family history was insignificant for any neurological disease. There was no parent consanguinity or gastrointestinal symptoms. Based on the clinical course of the patient, he was initially diagnosed as Friedreich ataxia. Neurological examination showed a broad‐based gait which was clearly ataxic. His broad‐based gait was associated with tongue fasciculation, intentional tremors, and dysdiadokokinesia. Upper and lower limb examination confirmed cerebellar ataxia with intention tremor and absent deep tendon reflexes. He was noted to have ankle clonus. Motor examination revealed decreased extremity strength with 3/5 in the lower extremities versus 3/5 in the upper extremities. Joint position sensation, vibration sense, sensitivity, and cranial nerve function were normal. No head titubation or abnormal movement was observed. Intellectual abilities were adequate for the age of the patient. All others to systemic examination were unremarkable. His height and weight were normal for his age group.

The results of the patient's routine blood chemistries, electrocardiography, echocardiography, brain magnetic resonance, visual evoked potentials, and troncular evoked auditory potentials had no pathologic alterations. Ophthalmologic examination revealed simple myopia.

Laboratory investigation and their result are interpreted in Table 1.

**TABLE 1:** Laboratory investigation

Investigation	Value	Normal range
Vitamin A	45	20–60 mcg/dl
Vitamin E	3.9	6–10 mg/dl
Creatinine kinase	80	10–120 mcg/l

Genetic analysis of the frataxin gene confirmed two alleles in the normal size range and no evidence of an expansion. Further investigation confirmed evidence of vitamin E deficiency. His vitamin E levels were 3.9 mg/dl. (Normal:6–10 mg/dl). Other laboratory investigations confirmed a normal full blood count, glucose, liver, kidney function, coeliac antibodies, fasting lipids, thyroid function tests, copper, and ceruloplasmin. Magnetic resonance imaging (MRI) of the brain confirmed normal intracranial appearances including no cerebellar atrophy. The diagnosis of AVED was confirmed by mutations in the TTPA gene. There was a homozygous pathogenic frame shift mutation in the TTPA gene *c.706del (p.[His236fs])*, which results in loss of activity of the *α*‐TTP. The patient was started treatment with high‐dose vitamin E in the form of D‐alpha‐tocopherol supplementation. Most importantly, neurologic symptoms improve when levels are normal, to our distress symptoms reappear when the level of vitamin E drop.

Treatment was started with 1800 mg/d oral vitamin E. After few weeks of vitamin E supplementation, his functional level was substantially improved. The muscle strengths of the bilateral upper and lower extremities were improved to 3/5 in the proximal muscles. He was able to gait on a walker with moderate assistance. He has less gait instability, his walk is almost normal (he joined a soccer team and danced at the end‐of‐school party), and his speech became more fluent. Manual abilities have improved but are not yet normal. His vitamin E level improved to 17.34 mg/dl. He was discharged from the hospital and on follow‐up, after a month he was improving and had no fresh issues.

## 3 DISCUSSION

We present the case of AVED patient in Nepal, which was first diagnosed as Friedreich ataxia before genetic tests were conducted. Ataxia can be due to inherited (genetic) or acquired (nongenetic) causes, and they must be distinguished for the definitive management of the ataxia. Many diagnostic modalities like molecular genetic testing along with family history and physical examination are needed to diagnose the genetic forms of ataxia.

The age of onset of the ataxia recessive cerebellar ataxia (ARCA) disorders is usually in childhood and teens, not after the age of 40. There are limited treatments available for these disorders but treatable causes of ataxia‐like ataxia with vitamin E deficiency, Refsum disease, and Niemann Picks disease need to be investigated to be treated with their available medication. Treatment should be focused to reduce a life span and improve the quality of life with increase mobility and communication of the individual.

**FIGURE 2:**
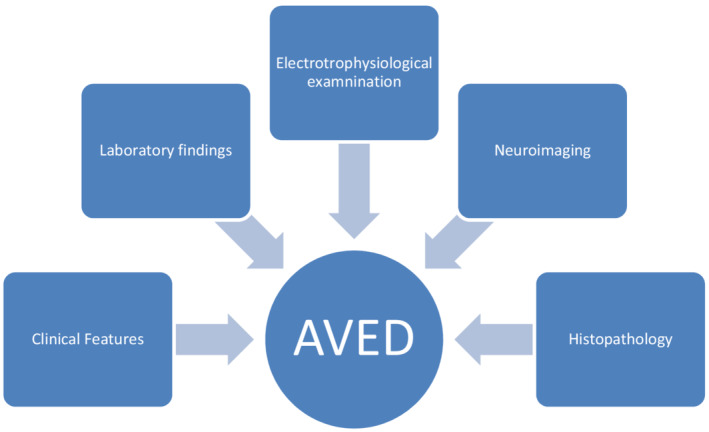
Method of diagnosis of ataxia due to vitamin E deficiency

Ataxia with vitamin E deficiency typically manifests in late childhood or early teens between ages 5 and 15 years. Progressive ataxia with the clumsiness of hands with decreased reflexes is the main characteristics symptoms of the AVED. No definitive and consensus diagnostic criteria exist at present for AVED. The following approach should be undertaken to diagnose the AVED. (Figure 2).

Clinical symptoms like progressive ataxia with decreased reflex remain the prominent symptoms of AVED. Laboratory findings include very low plasma vitamin E concentration, and the range usually depends on the method of performed test and varies among different laboratories. Lipid and lipoprotein profiles are usually within normal limits. There are no definitive electrophysiological findings specific to AVED. According to the study done in North Africa, most of the individuals with AVED had mild neuropathy and were mostly combined types of neuropathies (both sensory and motor).^4^ Marriotti et al.^5^ 2004 reported cerebellar atrophy in some individuals with AVED. Spinal sensory demyelination with neuronal atrophy and axonal spheroids and neuronal lipofuscin accumulation in the third cortical layer of the cerebral cortex, thalamus, lateral geniculate body, spinal horns, and posterior root ganglia are the commonest histopathology findings.^6^ The following criteria should be met to diagnose the AVED:

**Table:** 

Friedreich ataxia‐like neurologic phenotype
Markedly reduced plasma vitamin E
Normal lipoprotein profile
Exclusion of disease that cause fat malabsorption

Genetic counseling should be done to provide the individuals and families with information on nature, mode of inheritance, and the importance of vitamin E supplementation to pre‐symptomatic individuals to prevent them from having neurological symptoms.

**FIGURE 3:**
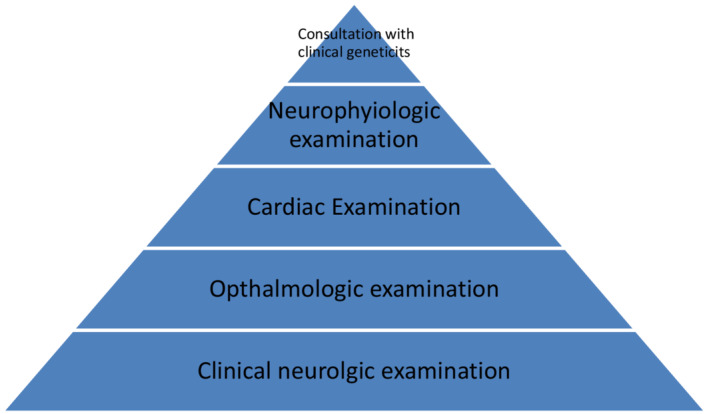
Evaluation of patient with AVED

The following mentioned evaluations should be done to know the extent of the disease in an individual diagnosed with AVED. (Figure 3) Clinical neurological examination to know the reflex status, and gait disturbances with Babinski sign should be accessed to know the extent of AVED. Ophthalmological examination includes examination for evidence of ocular degeneration or retinitis pigmentosa. Electrocardiogram (ECG) and echocardiography should be done to access the complication of AVED in the heart. Nerve conduction studies should be performed to know the status of neurologic improvement after vitamin E. Genetic counseling is preferred to make risk individuals prevent the effects of vitamin E.

High doses of vitamin E usually lead to the improvement in most neurological manifestations, but recovery is slow and incomplete.^7^ Vitamin E supplementation should be started as soon as possible, but the optimal dosage is still variable. The doses and the route of vitamin E supplementation depend on the causes of vitamin E deficiency. The treatment of choice for AVED is lifelong high‐dose oral vitamin E supplementation. The early use of vitamin E supplementation in the disease process helps to reverse some neurological symptoms like ataxia and intellectual deterioration.^8^ The prognosis despite the supplementation of vitamin E depends on the timing of the supplementation and the age of the onset of vitamin E deficiency. Older individuals usually remain deficient in proprioception and gait unsteadiness although the progression of the disease can be stopped with vitamin E supplementation.^4^ The ideal dose of vitamin E ranges from 800 mg to 1500 mg (40 mg/kg/day) in children.^5^, ^7^ Our patient was treated with vitamin E supplementation with a dose of 600 mg three times a day. Vitamin E treatment in pre‐symptomatic individuals with a history of family cases of AVED can help to prevent the individual from primary manifestations. The symptoms do not develop in the individuals with the initiation of vitamin E earlier in pre‐symptomatic individuals who are at risk.^9^

Periodic follow‐up is required during vitamin E therapy. The plasma concentration of vitamin E should be measured in regular intervals, usually, every 6 months to maintain the level of vitamin E in the high normal range. Individuals with AVED should avoid smoking and occupations requiring quick responses or good balance. Smoking reduces the total radical trapping antioxidant parameter of plasma (TRAP), which is regarded as the best prognostic marker during the supplementation of vitamin E, leading to the reduction in plasma vitamin level.^10^

The recommended doses of vitamin E for age group and associated causes are shown in Table 2.

**TABLE 2:** Recommended doses of vitamin E according to age group and associated causes

Age group	Associated symptoms	Dose and route of supplementation
Infants and children	Cholestasis	17–35 mg/kg/day of RRR alpha‐tocopherol (natural source of vitamin E). May increased to 70–130 mg/kg/day, to achieve normal serum measurements of vitamin E Water‐Miscible vitamin E, 10–17 mg/kg/day can be used^11^
Adults	Fat malabsorption	Variable doses of vitamin E −50 to 500 mg /day, then adjusted as needed to achieve normal serum measurements of vitamin E
Any age group	Severe cholestasis/genetic disorders related to transport of vitamin E	Not respond to oral supplementation Intramuscular vitamin E should be used if available and practical as it requires frequent weekly dosing^12^

## 4 CONCLUSION

Ataxia with vitamin E deficiency (AVED) is a rare cause of hereditary ataxia in developing countries with unknown prevalence. Vitamin E should always be assessed in progressive ataxia of genetic or unexplained causes and especially with Friedreich's ataxia‐like phenotype since treatment is available. Neurologists should keep AVED as their differential diagnosis in patients presenting with ataxia as AVED is a reversible disease if treated in time with vitamin E supplementation. Vitamin E supplementation improves symptoms and prevents the progression of the disease.

## AUTHOR CONTRIBUTIONS

ST wrote the original manuscript, reviewed, and edited the original manuscript. SS and SC reviewed and edited the original manuscript.

## CONFLICT OF INTEREST

None.

## ETHICAL APPROVAL

None.

## CONSENT

Written informed consent was obtained from the patient for publication of the report.

## Data Availability

All the required information is in manuscript itself.
